# Glycolysis Aids in Human Lens Epithelial Cells’ Adaptation to Hypoxia

**DOI:** 10.3390/antiox12061304

**Published:** 2023-06-19

**Authors:** Yuxin Huang, Xiyuan Ping, Yilei Cui, Hao Yang, Jing Bao, Qichuan Yin, Hailaiti Ailifeire, Xingchao Shentu

**Affiliations:** Eye Center, The Second Affiliated Hospital, School of Medicine, Zhejiang University, Zhejiang Provincial Key Laboratory of Ophthalmology, Zhejiang Provincial Clinical Research Center for Eye Diseases, Zhejiang Provincial Engineering Institute on Eye Diseases, Hangzhou 310009, China

**Keywords:** hypoxia, glycolysis, endoplasmic reticulum (ER) stress, reactive oxygen species (ROS), apoptosis

## Abstract

Hypoxic environments are known to trigger pathological damage in multiple cellular subtypes. Interestingly, the lens is a naturally hypoxic tissue, with glycolysis serving as its main source of energy. Hypoxia is essential for maintaining the long-term transparency of the lens in addition to avoiding nuclear cataracts. Herein, we explore the complex mechanisms by which lens epithelial cells adapt to hypoxic conditions while maintaining their normal growth and metabolic activity. Our data show that the glycolysis pathway is significantly upregulated during human lens epithelial (HLE) cells exposure to hypoxia. The inhibition of glycolysis under hypoxic conditions incited endoplasmic reticulum (ER) stress and reactive oxygen species (ROS) production in HLE cells, leading to cellular apoptosis. After ATP was replenished, the damage to the cells was not completely recovered, and ER stress, ROS production, and cell apoptosis still occurred. These results suggest that glycolysis not only performs energy metabolism in the process of HLE cells adapting to hypoxia, but also helps them continuously resist cell apoptosis caused by ER stress and ROS production. Furthermore, our proteomic atlas provides possible rescue mechanisms for cellular damage caused by hypoxia.

## 1. Introduction

The lens is an avascular, protein-rich, and naturally hypoxic tissue [1,2,3]. The oxygen content of the lens epithelium in the eye has been reported to be about 1% [4], which plays an important role in maintaining the transparency of the lens and avoiding nuclear cataracts [5]. Notably, such hypoxic conditions can inhibit cell proliferation and even cause pathological damage to various cell types, such as haematopoietic stem cells, keratinocytes, lymphocytes, and a wide variety of cancer cells [6]. Therefore, exploring the adaptation mechanism of the lens to hypoxia has become a current research hotspot.

Glycolysis is intricately linked to numerous pathological pathways in human physiology, such as malignancies, neurological disorders, metabolic disorders, and other epidemics [7,8,9,10,11]. Hypoxia inhibits the tricarboxylic acid (TCA) cycle, leaving glycolysis as the primary metabolic pathway responsible for converting glucose into usable energy in a process known as anaerobic glycolysis [12]. It has been reported that the lens has very low energy requirements under hypoxic conditions, utilising anaerobic glycolysis to obtain most of its ATP [13]. The long-term adaptation mechanism of tumour cells to hypoxia is through the HIF-1(hypoxia-inducible factor-1)-dependent upregulation of the synthesis of glucose transporters and glycolytic enzymes, thereby increasing glycolytic flux [14,15,16,17]. This hypoxia-induced increase in glycolytic flux favours cell survival during hypoxic injury and is therefore adaptive in nature [18]. Nonetheless, the precise function of glycolysis in the adaptation of lens epithelial cells to hypoxic environments remains incompletely understood.

The reprogramming of vital cellular processes (including gene expression, post-transcriptional and post-translational modification of gene products, mitochondrial respiration, and energy metabolism) serves as an integral means of coordinating cellular responses to hypoxia [19]. We speculated that human lens epithelial (HLE) cells survive under hypoxic conditions through complex signal transduction pathways. In this study, we used LC-MS/MS proteomics to explore the potentially adaptive mechanisms of HLE cells under hypoxia in addition to performing bioinformatics analysis and verification. Our study provides a proteomic map of HLE cell adaptation to hypoxia. At the same time, we found that glycolysis was not only an energy source, but also played a role in the process of cellular resistance to both oxidative stress and apoptosis.

## 2. Materials and Methods

### 2.1. Human Lens Epithelial (HLE) Cell Cultures and Treatment

The human lens epithelial (HLE) cell lines (SRA01-04, RIKEN Cell Bank, Tokyo, Japan) were maintained in DMEM (Corning, NY, USA) with 20% FBS (AusgeneX, Brisbane, QLD, Australia) and a 1% penicillin–streptomycin solution (Gibco, Carlsbad, CA, USA) in a 5% CO_2_ incubator at 37 °C. For the hypoxia culture, HLE cells were seeded onto a 6 cm Petri dish overnight and were then cultured in a hypoxia incubator (AW400SG, Electrotek, Shipley BD18 4EW, West Yorkshire, UK) with 1% O_2_, 5% CO_2_, and 94% N_2_ at 37 °C for 24 h. For 2-Deoxy-D-glucose (2-DG) treatment, the HLE cells were seeded onto a 6 cm Petri dish overnight and then treated with 2-DG (14 mM, HY-13966, MCE, Monmouth Junction, NJ, USA) before being cultured in a hypoxia incubator for 24 h. For ATP replenishment, the HLE cells were seeded onto a 6 cm Petri dish overnight, treated with 2-DG (14 mM, HY-13966, MCE, Monmouth Junction, NJ, USA) and ATP (12.5 μM, HY-B2176, MCE, Monmouth Junction, NJ, USA), and then cultured in a hypoxia incubator for 24 h.

### 2.2. Light Microscopy

After experimental treatment, the HLE cells were placed under a light microscope (Leica Microsystems Ltd.CH-9435, Heerbrugg, Switzerland) to observe their morphology.

### 2.3. Cell Counting Kit-8 (CCK8) Assay

A Cell Counting Kit-8 (CCK-8, Dojindo Molecular Technologies, Kyushu Island, Japan) was used to analyse and evaluate cell viability. HLE cells were seeded at 1 × 10^4^ cells/well in 96-well plates overnight. After experimental treatment, the medium was removed, and the cells were washed with phosphate-buffered saline (PBS). Each well was refilled with a fresh medium containing 10% CCK-8 reagent before being placed in the cell incubator/hypoxia incubator for another 2 h. Finally, a 96-well microplate reader (Bio-Rad, Hercules, CA, USA) was used to evaluate cell viability at an optical density of 450 nm.

### 2.4. Liquid Chromatography-Tandem Mass Spectrometry (LC-MS/MS) Proteomics

Extraction of protein and peptide segment enzymolysis: The HLE cells were seeded in three 10 cm Petri dishes overnight before being treated for 24 h under either hypoxic or normoxic conditions. The treated cells were collected as samples; protein was extracted using the SDT (4% SDS, 100 mM Tris-HCl, 1 mM DTT, pH 7.6) lysis method before being quantified with a BCA Protein Assay Kit (Bio-Rad, Hercules, CA, USA). Protein digestion with trypsin was performed in accordance with the filter-aided sample preparation (FASP) procedure described by Matthias Mann. The digest peptides of each sample were desalted on C18 Cartridges (Empore SPE Cartridges C18 (standard density), bed I.D. 7 mm, volume 3 mL, Sigma), concentrated using vacuum centrifugation, and reconstituted in 40 µL of 0.1% (*v*/*v*) formic acid.

Tandem Mass Tag (TMT) Labelling and High pH Reversed-Phase Peptide Fractionation: We labelled 100 μg of the peptide mixture of each sample using TMT reagent according to the manufacturer’s instructions (Thermo Fisher Scientific, Waltham, MA, USA). The labelled peptides were fractionated using a High pH Reversed-Phase Peptide Fractionation Kit (Thermo Fisher Scientific).

LC-MS/MS Data Acquisition: LC-MS/MS analysis was performed on a Q Exactive mass spectrometer (Thermo Fisher Scientific) that was coupled to an Easy nLC (Proxeon Biosystems, now Thermo Fisher Scientific) for 60/90 min.

Identification and quantitative analysis of proteins: The UniProt database was used in this experiment. The MS raw data for each sample were searched using the MASCOT engine (Matrix Science, London, UK; version 2.2) embedded into Proteome Discoverer 1.4 software for identification and quantitation analysis. Relevant parameters and instructions are as follows: Max Missed Cleavages, 2; Peptide Mass Tolerance, ±20 ppm; Fragment Mass Tolerance, 0.1 Da; Database schema used to calculate FDR, Decoy; Peptide FDR, ≤0.01.

Bioinformatic Analysis and Visualisation: The quantitative information of the target protein set was normalised (normalised to the (−1, 1) interval). The OECloud tools at https://cloud.oebiotech.com (accessed on 18 January 2023) were used to analyse differentially expressed proteins and protein expression levels and to generate volcano maps and heat maps. GO and KEGG (databases: KEGG PATHWAY, Gene Ontology) analyses were conducted to analyse the protein families and pathways in each group. Visualisation was performed using the Cloud tools at https://hiplot.com.cn (accessed on 18 January 2023). 

### 2.5. Western Blotting Analysis

After treatment, the HLE cells were lysed in RIPA buffer containing protease inhibitors and phosphatase inhibitors. The concentration of the obtained protein was measured using a BCA Protein Assay Kit (Cat. No. BL521A, Beijing Labgic Technology Co., Ltd, Beijing, China). The protein samples were then resolved in 1 × SDS loading buffer and heated at 95 °C for 5–10 min. An equal quantity of samples was electrophoresed using a 4–20% SDS-PAGE gel and then transferred onto 0.22 µm PVDF membranes. Quick Blocking Buffer (Cat. No. P30500, New Cell&Molecular Biotech Co., Ltd, Jiangsu, China) was used as a blocking agent for 10 min, and then the PVDF membranes were incubated with primary antibodies overnight at 4 °C. The PVDF membranes were washed in 1× TBST three times (10 min each) and incubated with the HRP-coupled secondary antibody for 1–2 h. The protein bands were visualised using the SuperSignal Western Blot Substrate bundle (Pierce, Thermo Fisher Scientific). The data were analysed using Image Lab software (version 6.1; Hong Kong Bio-Rad). The following antibodies were used for immunoblots: anti-β-actin (HRP-60008, Proteintech, Rosemont, IL, USA), anti-HK1 (2024, Cell Signaling Technology (CST), Danvers, MA, USA), anti-HK2 (sc-374091, Santa Cruz Biotechnology, Santa Cruz, CA, USA), anti-PGK1 (sc-130335, Santa Cruz Biotechnology), anti-ENO2 (65162, CST), anti-LDHA (3582, CST), anti-GRP78 (sc-166490, Santa Cruz Biotechnology), anti-CHOP (sc-7351, Santa Cruz Biotechnology), anti-XBP1 (sc-8015, Santa Cruz Biotechnology), anti-CASP3 (sc-7272, Santa Cruz Biotechnology), anti-CASP4 (sc-56056, Santa Cruz Biotechnology), anti-CASP9 (sc-56076, Santa Cruz Biotechnology), anti-CYCS (11940, CST), anti-NRF2 (80593-1-RR, Proteintech), and anti-NOX4 (ET1607-4, HUABIO, Hangzhou, China).

### 2.6. Quantitative Real-Time Polymerase Chain Reaction Analysis

We used 1 mL of Trizol reagent to extract the total RNA and transcribed 1000 ng of RNA into cDNA. Quantitative real-time polymerase chain reaction (Q-PCR) was conducted with a ChamQ Universal SYBR qPCR Master Mix kit (Q711-02, Vazyme, Nanjing, China) and a 7500 Fast Real-Time PCR System. The sequence of primers is summarised in Table S1. The relative RNA levels were analysed using the 2^−ΔΔCt^ quantification method after normalisation to β-actin.

### 2.7. Immunofluorescence

After washing with PBS, the cells were fixed in 4% paraformaldehyde solution for 15 min and blocked with 10% goat serum albumin for 1 h, followed by incubating with primary antibodies overnight at 4 °C, after which they were stained with secondary antibodies and DAPI for 1 h at room temperature. The primary and secondary antibodies used were as follows: anti-GRP78 (1:50, sc-166490, Santa Cruz Biotechnology), anti-CHOP (1:50, sc-7351, Santa Cruz Biotechnology), anti-XBP1 (1:50, sc-8015, Santa Cruz Biotechnology), Alexa Fluor 488 (1:1000; A28175, Thermo Fisher Scientific), and Alexa Fluor 555 (1:1000; A21428, Thermo Fisher Scientific). After washing three times, the cells were observed using a Nikon A1 confocal microscope (Tokyo, Japan).

### 2.8. Reactive Oxygen Species (ROS) Measurement

The intracellular ROS level was measured using a 2′,7′-dichlorofluorescein diacetate probe (DCFH-DA, Beyotime Institute of Biotechnology, Shanghai, China). After treatment, the HLE cells were incubated with a serum-free culture medium containing 10 mM DCFH-DA probe in a cell incubator for 20 min. The images of intracellular ROS were captured using a fluorescence microscope (Leica DMi8) within 15 min after being washed three times with a serum-free culture medium.

### 2.9. Determination of Apoptosis

To evaluate apoptosis, after treatment, the HLE cells were determined by using the Annexin V–apoptosis detection kit (Beyotime Institute of Biotechnology) according to the manufacturer’s instructions. The cells were incubated with Annexin-V–FITC and propidium iodide (PI) for 10 min, after which images of the intracellular Annexin V-FITC were captured using a fluorescence microscope (Leica DMi8).

### 2.10. Statistical Analysis

The data were analysed using GraphPad Prism 8.0 software and are presented as means ± SD. Student’s *t*-tests were performed to compare the statistical differences between two groups, and one-way ANOVA was conducted to compare the data of multiple groups. A value of *p* < 0.05 was considered significant.

## 3. Results

### 3.1. Hypoxia Treatment Does Not Alter the Cell Morphology and Viability of HLE Cells

**Figure 1:**
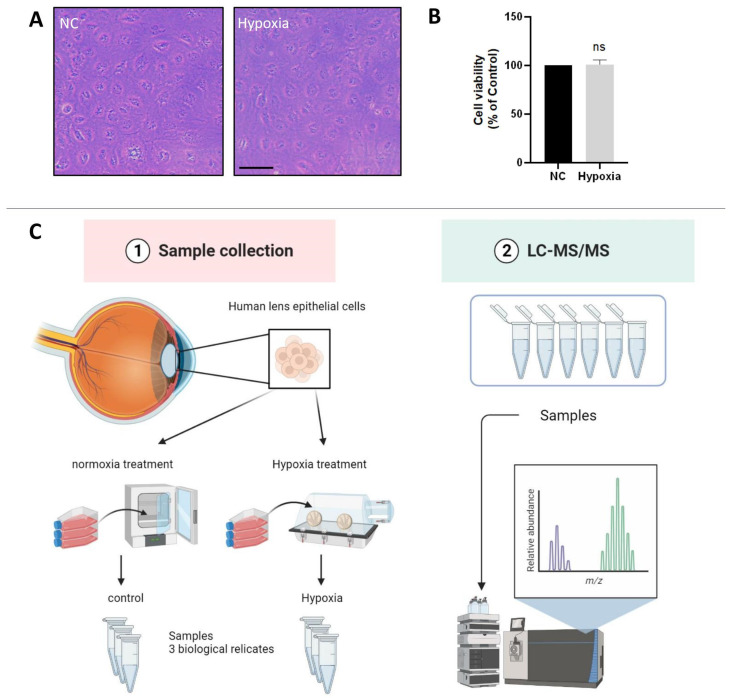
**HLE cells adapt to a hypoxic environment.** (**A**) Light photomicrographs of HLECs under hypoxic and normoxic conditions. Scale bars: 500 μm. (**B**) CCK8 assay was used to detect the cell viability, and the optical density (OD) value of the hypoxia and normoxia conditions showed no statistically significant change. Cell viability is expressed as the mean ± standard deviation of three independent experiments (ns: *p* > 0.05). (**C**) Schematic diagram of the proteomics process.

The oxygen content of the lens epithelium in the eye has been reported to be about 1% [5]. To investigate whether HLE cells adapt to a hypoxic environment, we measured the cell viability of HLE cells under hypoxic and normoxic conditions. The light microscopic images showed no abnormality in cell morphology or change in cell density of HLE cells under hypoxia conditions compared with normoxia (Figure 1A). As shown in Figure 1B, compared with the control group, there was no significant change in cell viability after 24 h of hypoxia treatment. This finding indicates that HLE cells can adapt to a hypoxic environment.

### 3.2. Proteomic Profiles of HLE Cells under Hypoxia and Normoxia Conditions

**Figure 2:**
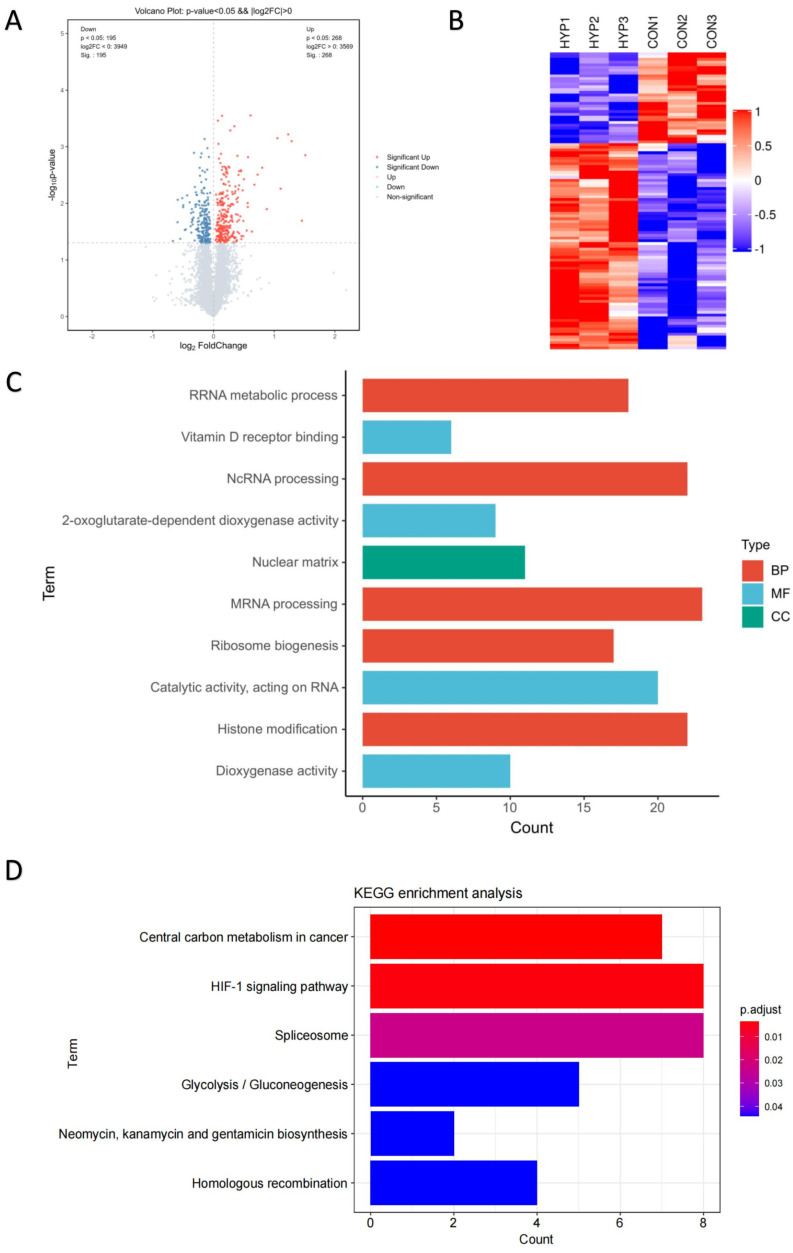
**Proteomic profiles of HLE cells under hypoxia and normoxia conditions.** TMT-based proteomic analysis on HLECs subjected to either hypoxia (HYP) or normoxia (CON) conditions. (**A**) Volcano analysis representing the significantly differentially expressed proteins (DEPs). (**B**) The heatmap demonstrates that expression patterns were altered under hypoxia. Each column represents a sample, and each row represents the expression levels of a single proteomic in various samples. The colour scale of the heatmap ranges from blue (low expression) to red (high expression). Top 10 enrichment analysis of GO terms for the differentially expressed proteins. The upregulated protein terms (**C**) in the biological process (BP), cellular component (CC), and molecular function (MF) categories are depicted. (**D**) KEGG based on upregulated differentially expressed proteins (DEPs) annotated based on proteomic analysis.

To reveal the molecular mechanism of HLE cells adapting to a hypoxic environment, the proteomic profiles of HLE cells under hypoxia and normoxia conditions were identified (Figure 1C). The volcano plot displays these differentially expressed proteins according to their fold changes and *p* values (Figure 2A). A total of 7518 proteins were identified in the six samples. A total of 463 of these proteins were significantly differentially expressed proteins (DEPs) (adjusted *p* < 0.05, |log2FoldChange| > 0). Among these DEPs, 268 were upregulated and 195 were downregulated, as demonstrated by heat map analysis (Figure 2B). 

With Gene Ontology and KEGG enrichment analysis, we performed an in-depth investigation of the molecular mechanisms of the proteome. Figure 2C shows the Top 10 GO pathways of the significantly upregulated proteins. RNA splicing, which occurs in the nucleus, processes mRNA and non-coding RNA through the spliceosome to generate mature mRNA and translate it into proteins [20,21]. During hypoxia, the expression of splicing factors can be observed, and the spliceosome can regulate many key cellular processes [21,22,23]. Recent studies have shown that 2-oxoglutarate-dependent dioxygenases (2-OGDs) can control the level and function of HIF during hypoxia [24], while dioxygenases are involved in almost all aspects of gene regulation, including chromatin organisation, transcription, and translation [25]. Hypoxia also affects the activity and gene expression of histone-modifying enzymes, thereby controlling the post-translational modification of histones and HIF [26].

Significantly upregulated proteins were enriched in six KEGG pathway terms with statistical significance (adjusted *p* < 0.05) (Figure 2D). The six KEGG pathway terms included central carbon metabolism in cancer; the HIF-1 signalling pathway; the spliceosome; glycolysis/gluconeogenesis; neomycin, kanamycin, and gentamicin biosynthesis; and homologous recombination. Emerging evidence suggests that the expression levels of key genes in the homologous recombination (HR) pathway in cancer cells are cooperatively repressed by hypoxia, leading to genetic instability [27]. In contrast, the HR pathway was upregulated in HLE cells, implying that the genetic stability of our HLE cells was not affected by hypoxia. The long-term adaptation mechanism of tumour cells to hypoxia is through the HIF-1-dependent upregulation of the synthesis of glucose transporters and glycolytic enzymes, thereby increasing glycolytic flux [14,15,16,17]. Therefore, we further explored the HIF-1 signalling pathway and the glycolysis pathway.

### 3.3. Validation of Glycolysis Pathway

**Figure 3:**
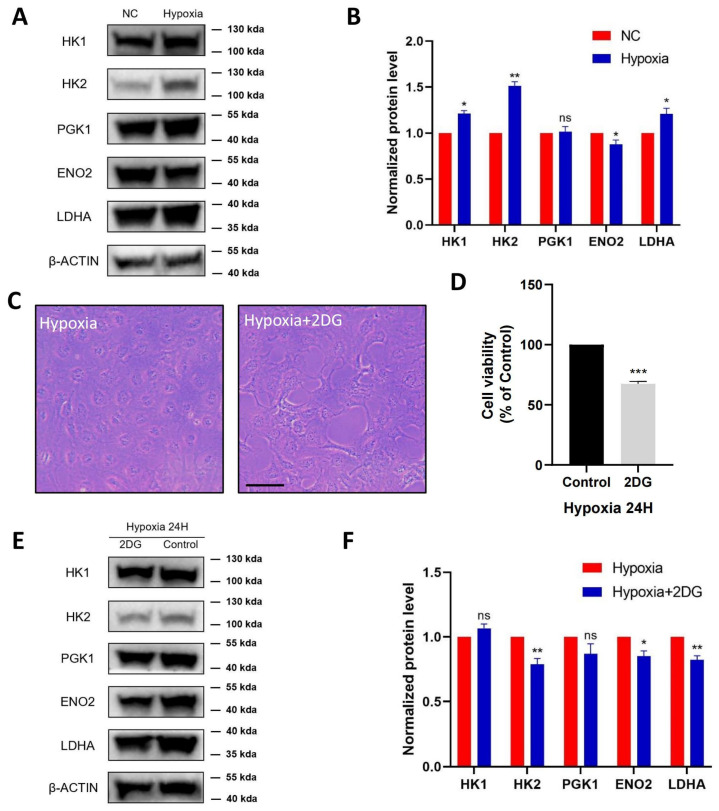
**Validation of glycolysis pathway in HLE cells.** Images represent protein levels of glycolysis-related proteins in HLECs under hypoxia and normoxia conditions. The protein levels of HK1, HK2, PGK1, ENO2, and LDHA were analysed via Western blot (**A**). (**B**) Bar graph shows quantification. Results are combined data from four experiments with different cell preparations, and each value represents mean ± SEM; *: *p* < 0.05, **: *p* < 0.01, ns: *p* > 0.05. (**C**) Light photomicrographs of HLECs cultured with or without glycolysis inhibitor 2DG under hypoxia conditions. Scale bars: 500 μm. (**D**) CCK8 assay was used to detect the cell viability and the optical density (OD) values of HLECs; those cultured with glycolysis inhibitor 2DG have significantly lower values under hypoxia conditions. Images represent protein levels of glycolysis-related proteins in HLECs cultured with or without glycolysis inhibitor 2DG under hypoxia conditions (***: *p <* 0.001). The protein levels of HK1, HK2, PGK1, ENO2, and LDHA were analysed via Western blot (**E**). (**F**) Bar graph shows quantification. Results are combined data from four experiments with different cell preparations, and each value represents mean ± SEM; *: *p* < 0.05, **: *p* < 0.01, ns: *p* > 0.05.

According to the proteomic profiles, we identified that HK1, HK2, PGK1, ENO2, and LDHA, serving as key enzymes of glycolysis, were jointly involved in the HIF-1 signalling pathway and glycolysis pathway. Therefore, we further verified these proteins in HLE cells using protein blot analysis (Figure 3A). Compared with the normoxia group, HK1, HK2, and LDHA were significantly upregulated in the hypoxia group with statistical significance, ENO2 was significantly downregulated, and PGK1 was not significantly changed (Figure 3B). 

To further investigate the regulation of glycolysis in HLE cells under hypoxia conditions, HLE cells were treated with the glycolysis inhibitor 2-Deoxy-D-glucose (2DG). As shown in (Figure 3C), in hypoxia-treated HLE cells, the addition of 2DG changed cell morphology and inhibited cell proliferation, resulting in a significant decrease in the cell viability of the HLE cells (Figure 3D). Western blotting showed that the protein levels of glycolysis-related proteins HK2, ENO2, and LDHA were significantly downregulated in HLE cells treated with the glycolysis inhibitor 2DG under hypoxia conditions (Figure 3E,F).

### 3.4. Inhibition of Glycolysis Induced ROS, ER Stress, and Apoptosis

**Figure 4:**
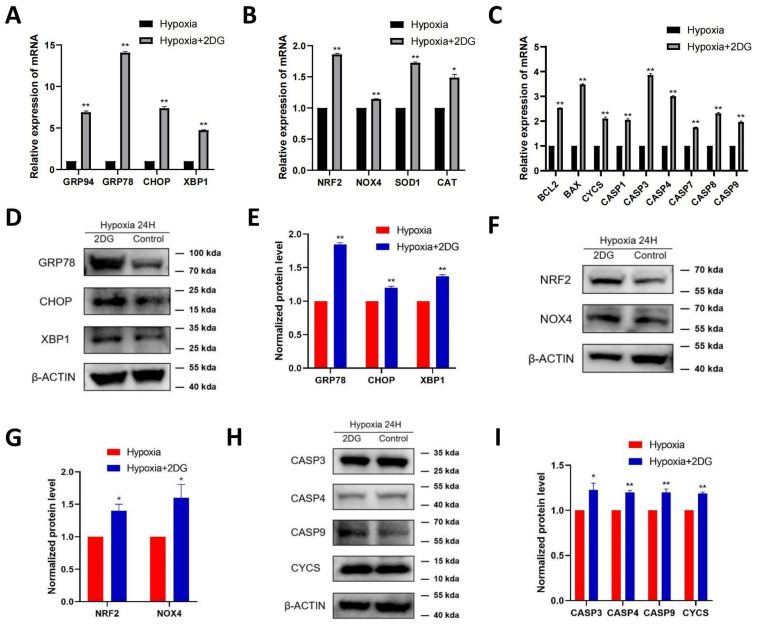
**Inhibition of glycolysis induced the upregulation of ROS-, ER stress-, and apoptosis-related proteins.** The expression of ER stress markers (GRP94, GRP78, CHOP, XBP1), ROS markers (NRF2, NOX4, SOD1, CAT), and apoptosis markers (BCL2, BAX, CYCS, CASP1, CASP3, CASP4, CASP7, CASP8, CASP9) in HLECs was measured after culturing with or without glycolysis inhibitor 2-deoxyglucose (2DG) under hypoxia conditions by qRT-PCR (**A**–**C**). Images represent protein levels of ER stress markers, apoptosis markers, and ROS markers in HLECs cultured with or without glycolysis inhibitor 2-deoxyglucose (2DG) under hypoxia conditions (**D**,**F**,**H**). The protein levels of GRP78, CHOP, XBP1, NRF2, NOX4, CASP3, CASP4, CASP9, and CYCS were analysed via Western blot; bar graph shows quantification (**E**,**G**,**I**). Results are combined data from four experiments with different cell preparations, and each value represents mean ± SEM; *: *p* < 0.05, **: *p* < 0.01.

**Figure 5:**
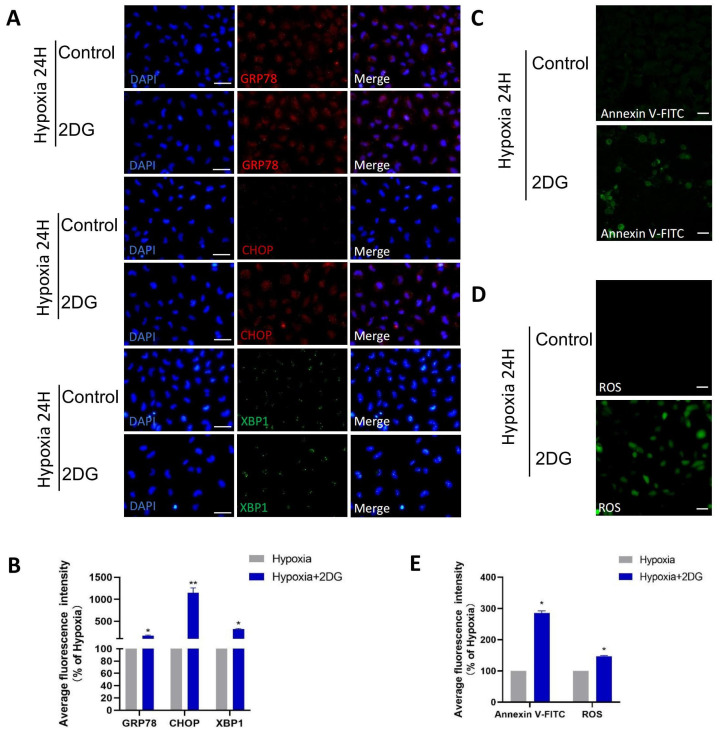
**Inhibition of glycolysis induced ROS, ER stress, and apoptosis.** (**A**) Representative immunofluorescence images of GRP78 (red), CHOP (red), XBP1 (green), and DAPI (blue) in HLE cells treated or untreated with 2DG under a hypoxia environment (magnification 40×). Scale bars: 50 μm. (**B**) The average fluorescence intensities of GRP78, CHOP, and XBP1 in immunofluorescence experiments (*n* = 3, >30 cells per experiment, *: *p* < 0.05, **: *p* < 0.01). (**C**,**D**) Representative fluorescence images depicting the intracellular Annexin V-FITC and ROS levels of HLE cells treated or untreated with 2DG under a hypoxia environment (magnification 40×). Scale bars: 100 μm. (**E**) The average fluorescence intensities of Annexin V-FITC and ROS in HLE cells (*n* = 3, >30 cells per experiment, *: *p* < 0.05).

In HLE cells treated with 2DG under hypoxia conditions, we found upregulated expression of endoplasmic reticulum (ER) stress markers [28], reactive oxygen species (ROS) markers [29,30], and apoptosis markers [31,32,33] as measured using qRT-PCR and Western blot (Figure 4A–C). The results of qRT-PCR show that the mRNA levels of ER stress markers (GRP94, GRP78, CHOP, XBP1), ROS markers (NRF2, NOX4, SOD1, CAT), and apoptosis markers (BCL2, BAX, CYCS, CASP1, CASP3, CASP4, CASP7, CASP8, CASP9) were significantly upregulated (Figure 4A–C). Western blot analysis showed that the protein levels of GRP78, CHOP, XBP1, NRF2, NOX4, CASP3, CASP4, CASP9, and CYCS were significantly upregulated (Figure 4D–I). The results of immunofluorescence also show that the average fluorescence intensities of the ER stress markers GRP78, CHOP, and XBP1 were significantly enhanced (Figure 5A,B). Likewise, we also observed that ROS and apoptosis occurred after the addition of 2DG relative to the hypoxia control group (Figure 5C–E). Thus, inhibition of glycolysis leads to the upregulation of ER stress, ROS, and apoptosis in HLE cells under hypoxia.

### 3.5. Replenishing ATP Did Not Rescue Damage Caused by Glycolysis Inhibition in HLE Cells 

**Figure 6:**
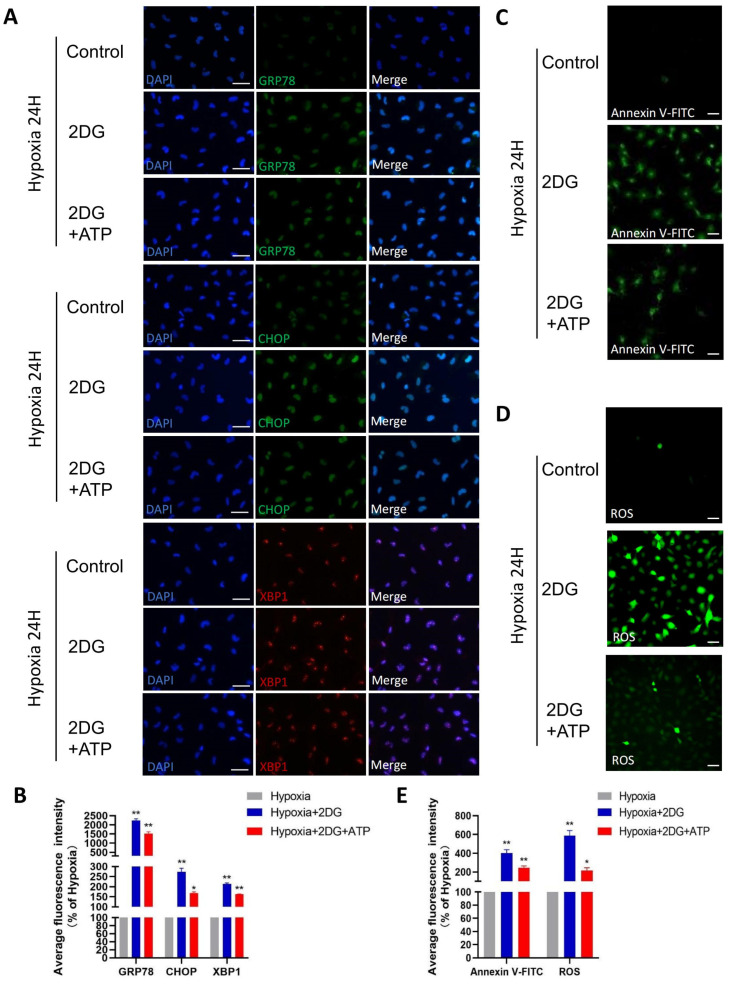
**Replenishing ATP did not rescue damage caused by glycolytic inhibition in HLE cells.** (**A**) Representative immunofluorescence images of GRP78 (green), CHOP (green), XBP1 (red), and DAPI (blue) in HLE cells treated with 2DG/2DG + ATP or untreated under a hypoxia environment (magnification 40×). Scale bars: 50 μm. (**B**) The average fluorescence intensities of GRP78, CHOP, and XBP1 in immunofluorescence experiments (*n* = 3, >30 cells per experiment, *: *p* < 0.05, **: *p* < 0.01). (**C**,**D**) Representative fluorescence images depicting the intracellular Annexin V-FITC and ROS levels of HLE cells treated with 2DG/2DG + ATP or untreated with 2DG under a hypoxia environment (magnification 40×). Scale bars: 100 μm. (**E**) The average fluorescence intensities of Annexin V-FITC and ROS in HLE cells (*n* = 3, >30 cells per experiment, *: *p* < 0.05, **: *p* < 0.01).

In lens epithelial cells, anaerobic glycolysis is the main source of bioenergy [34,35]. Given that the role of glycolysis is providing the main energy for HLE cells in a hypoxic environment, we replenished ATP while inhibiting glycolysis to explore the possible changes in HLE cells. When ATP is replenished while glycolysis is inhibited, ER stress, ROS, and apoptosis still occur in HLE cells, but these responses are attenuated compared to no ATP replenishment (Figure 6A–E). These results suggest that glycolysis is not only the main energy source but also plays other roles in the adaptation of HLE cells to hypoxia.

## 4. Discussion

A hypoxic environment is essential to maintain the normal physiological function of lens epithelial cells and lens transparency [5,36,37]. Consequently, the destruction of this environment causes the apoptosis of HLE cells, resulting in cortical cataracts [38,39]. Our results consistently indicate that HLE cells can adapt well to a hypoxic environment. Similarly, there are also cell types such as tumour cells and intestinal epithelial cells that can survive long-term under hypoxic conditions [40,41]. To further explore how they sense and adapt to oxygen supply, our study performed proteomic detection and analysis on HLE cells in a hypoxic environment.

Our study detected significant upregulation of the HIF-1 signalling pathway and glycolysis pathway, playing integral roles in lens response to hypoxia and energy supply, respectively [35,38,42,43]. We detected the co-enriched proteins HK1, HK2, PGK1, ENO2, and LDHA in the HIF-1 signalling pathway and the glycolysis pathway. Meanwhile, these five proteins serve as key enzymes of glycolysis. Cells in a hypoxic environment have a strong ability to adapt to O2 deprivation [18], mainly sensing hypoxia through hypoxia-inducible factor (HIF) and regulating the expression of a series of downstream genes to participate in multiple processes, such as cell metabolism, cell growth, cell proliferation, and glycolysis [6]. Accumulating evidence has shown that hexokinases play an important role not only in glycolysis but also in cell survival [44,45,46,47,48]. LDHA is involved in glycolysis, gene transcription, cell cycle regulation, and apoptosis [49,50,51]. ENO2 could affect the malignant biological behaviour of cancer cells [52,53]. Hence, additional roles of these proteins in HLE cell adaptation to hypoxia remain to be explored.

In specific pathological contexts, impaired glycolysis can trigger the activation of metabolic mechanisms aimed at compensating for the shortage of ATP and consequential rise in oxidative stress, cellular dysfunction, and loss of cellular vitality [54,55]. Our research observed that inhibition of glycolysis during hypoxic conditions in HLE cells caused endoplasmic reticulum stress, as well as reactive oxygen species production and apoptosis. However, the above situation still occurred after ATP supplementation. It has been shown that enhancing glycolysis in multiple cell models prevents cell damage and apoptosis by reducing ROS production [56,57]. These findings suggest that glycolysis in HLE cells may have additional crucial roles other than simply supplying energy during cellular adaptation to hypoxic conditions.

Cataracts caused by all environmental stressors involve endoplasmic reticulum stress, which generates severe levels of ROS, thereby inducing apoptosis of LECs [34,38,58]. The antioxidant defence system composed of the tear film, cornea, and aqueous humour is essential for maintaining redox homeostasis and preventing oxidative damage in the eye [59]. Recent observations led to the suggestion that the vitreous body may protect the lens from exposure to excess oxygen [60,61]. A study discussed the possibility that the chaperones αA and αB-crystallin are important cellular defence mechanisms in lens epithelial cells [5]. Our study identified the importance of glycolysis for the maintenance of redox homeostasis in human lens epithelial cells. Interference with the glycolytic process could potentially lead to oxidative stress in the lens and subsequently instigate cataract formation.

Alongside glucose catabolism and ATP generation, glycolysis also generates various consequential metabolites pivotal to cellular upkeep while participating in other cellular activities [62,63,64,65]. Hence, further investigation into the diverse functions of glycolysis in the adaptation of HLE cells to hypoxia can be pursued subsequently. Alterations in the folding pathway led to the accumulation of misfolded and unfolded proteins in the ER lumen, resulting in ER stress. This triggers the production of ROS, which in turn can promote ER stress [66,67]. ROS formation and buildup have long been found to play a vital role in mediating programmed cell death (PCD) (apoptosis or even necrosis) at a moderately high concentration among different cell types [68]. Additionally, in HLECs, the ER stress markers (GRP78, CHOP, XBP1), ROS markers (NRF2, NOX4), and apoptosis markers (CYCS, CASP3, CASP4, CASP9) were found to be upregulated. The specific relationship among them needs to be further studied in the future.

Within this research, we probed proteomic alterations in HLE cells during adaptation to hypoxia via LC-MS/MS proteomics and explored the role of the glycolysis pathway. Inhibition of glycolysis induces endoplasmic reticulum stress and ROS generation leading to apoptosis in HLE cells. Replenishment of ATP can alleviate the degree of cell damage to a certain extent but not completely rescue it. Thus, we hypothesised that the enhancement of the glycolysis pathway not only supplies energy for human lens epithelial cells to sustain cellular proliferation and metabolic processes under hypoxic conditions, but also potentially confers the ability of these cells to persistently evade apoptosis arising from ER stress and ROS generation within the lens. Significantly, additional potential functions of glycolysis in the acclimatisation of HLE cells to hypoxia have yet to be comprehensively probed. Furthermore, our proteomic atlas provides possible rescue mechanisms for cellular damage caused by hypoxia.

## 5. Conclusions

In conclusion, this study focused on the role of glycolysis in the adaptation of human lens epithelial cells to hypoxia environments. Glycolysis not only performs energy metabolism during the adaptation of HLE cells to hypoxia, but also helps them sustainably resist apoptosis caused by ER stress and ROS production. Our findings provide new clues on how cells adapt to hypoxia environments, and glycolysis may be a potential research direction in oxidative-stress-induced cataracts.

## Data Availability

The mass spectrometry proteomics data were deposited to the ProteomeXchange Consortium (http://proteomecentral.proteomexchange.org (accessed on 10 April 2023)) via the iProX partner repository [69,70] with the dataset identifier PXD041436.

## Supplementary Materials

The following supporting information can be downloaded at: https://www.mdpi.com/article/10.3390/antiox12061304/s1, Table S1: The sequence of qRT-PCR primers.
