# Supplementary material for: Glycolysis Aids in Human Lens Epithelial Cells’ Adaptation to Hypoxia
Source: Antioxidants (Basel). 2023 Jun 19;12(6):1304. doi: 10.3390/antiox12061304 (PMC10295312; doi:10.3390/antiox12061304)
Supplement: Supplementary file 1 [file antioxidants-12-01304-s001.zip › antioxidants-2366565-supplementary.pdf]

Supplementary Data

Table S1. The sequence of qRT-PCR primers

F: Forward Primer<br>R: Reverse Primer	Sequence (5' -> 3')	GenBank Accession
human-GRP94-F	CCAGTTTGGTGTCGGTTTCTAT	NM_003299
human-GRP94-R	CTGGGTATCGTTGTTGTGTTTTG	NM_003299
human-GRP78-F	CATCACGCCGTCCTATGTCG	NM_005347
human-GRP78-R	CGTCAAAGACCGTGTTCTCG	NM_005347
human-CHOP-F	GGAAACAGAGTGGTCATTCCC	NM_001195055
human-CHOP-R	CTGCTTGAGCCGTTCATTCTC	NM_001195055
human-XBP1-F	CCCTCCAGAACATCTCCCCAT	NM_001079539
human-XBP1-R	ACATGACTGGGTCCAAGTTGT	NM_001079539
human-NRF2-F	TCCAGTCAGAAACCAGTGGAT	NM_001145412
human-NRF2-R	GAATGTCTGCGCCAAAAGCTG	NM_001145412
human-NOX4-F	CAGATGTTGGGGCTAGGATTG	NM_001143836
human-NOX4-R	GAGTGTTCGGCACATGGGTA	NM_001143836
human-CAT-F	TGGGATCTCGTTGGAAATAACAC	NM_001752
human-CAT-R	TCAGGACGTAGGCTCCAGAAG	NM_001752
human-SOD1-F	GGTGGGCCAAAGGATGAAGAG	NM_000454
human-SOD1-R	CCACAAGCCAAACGACTTCC	NM_000454
human-BCL2-F	GGTGGGGTCATGTGTGTGG	NM_000657
human-BCL2-R	CGGTTCAGGTACTCAGTCATCC	NM_000657
human-BAX-F	CCCGAGAGGTCTTTTTCCGAG	NM_138763
human-BAX-R	CCAGCCCATGATGGTTCTGAT	NM_138763
human-CYCS-F	CTTTGGGCGGAAGACAGGTC	NM_018947
human-CYCS-R	TTATTGGCGGCTGTGTAAGAG	NM_018947
human-CASP1-F	TTTCCGCAAGGTTCGATTTTCA	NM_033294
human-CASP1-R	GGCATCTGCGCTCTACCATC	NM_033294
human-CASP3-F	CATGGAAGCGAATCAATGGACT	NM_004346
human-CASP3-R	CTGTACCAGACCGAGATGTCA	NM_004346
human-CASP4-F	CAAGAGAAGCAACGTATGGCA	NM_033306
human-CASP4-R	AGGCAGATGGTCAAACTCTGTA	NM_033306
human-CASP7-F	AGTGACAGGTATGGGCGTTC	NM_033339
human-CASP7-R	CGGCATTTGTATGGTCCTCTT	NM_033339
human-CASP8-F	GGGTCATCCTGGGAGAAGGA	NM_001137667
human-CASP8-R	ACAACTCCTCCCCTTTGCTG	NM_001137667
human-CASP9-F	CTCAGACCAGAGATTCGCAAAC	NM_032996
human-CASP9-R	GCATTTCCCCTCAAACTCTCAA	NM_032996
human-Bactin-F	CATGTACGTTGCTATCCAGGC	NM_001101
human-Bactin-R	CTCCTTAATGTCACGCACGAT	NM_001101
